# Carbon and nitrogen isotope fractionation of amino acids in an avian marine predator, the gentoo penguin (*Pygoscelis papua*)

**DOI:** 10.1002/ece3.1437

**Published:** 2015-02-25

**Authors:** Kelton W McMahon, Michael J Polito, Stephanie Abel, Matthew D McCarthy, Simon R Thorrold

**Affiliations:** 1 Ocean Sciences Department, University of CaliforniaSanta Cruz, California, 95064; 2 Biology Department, Woods Hole Oceanographic InstitutionWoods Hole, Massachusetts, 02543; 3 Department of Oceanography and Coastal Sciences, Louisiana State UniversityBaton Rouge, Louisiana, 70803; 4 Omaha's Henry Doorly Zoo and AquariumOmaha, Nebraska, 68107

**Keywords:** Amino acid, avian, compound-specific stable isotope analysis, diet, fractionation, penguin, trophic position

## Abstract

Compound-specific stable isotope analysis (CSIA) of amino acids (AA) has rapidly become a powerful tool in studies of food web architecture, resource use, and biogeochemical cycling. However, applications to avian ecology have been limited because no controlled studies have examined the patterns in AA isotope fractionation in birds. We conducted a controlled CSIA feeding experiment on an avian species, the gentoo penguin (*Pygoscelis papua*), to examine patterns in individual AA carbon and nitrogen stable isotope fractionation between diet (D) and consumer (C) (Δ^13^C_C-D_ and Δ^15^N_C-D_, respectively). We found that essential AA *δ*^13^C values and source AA *δ*^15^N values in feathers showed minimal trophic fractionation between diet and consumer, providing independent but complimentary archival proxies for primary producers and nitrogen sources respectively, at the base of food webs supporting penguins. Variations in nonessential AA Δ^13^C_C-D_ values reflected differences in macromolecule sources used for biosynthesis (e.g., protein vs. lipids) and provided a metric to assess resource utilization. The avian-specific nitrogen trophic discrimination factor (TDF_Glu-Phe_ = 3.5 ± 0.4‰) that we calculated from the difference in trophic fractionation (Δ^15^N_C__-D_) of glutamic acid and phenylalanine was significantly lower than the conventional literature value of 7.6‰. Trophic positions of five species of wild penguins calculated using a multi-TDF_G__lu-Phe_ equation with the avian-specific TDF_G__lu-Phe_ value from our experiment provided estimates that were more ecologically realistic than estimates using a single TDF_G__lu-Phe_ of 7.6‰ from the previous literature. Our results provide a quantitative, mechanistic framework for the use of CSIA in nonlethal, archival feathers to study the movement and foraging ecology of avian consumers.

## Introduction

Resource acquisition and allocation are fundamental requirements for all animals and significantly influence the behavior of individuals, the ecological and evolutionary trajectories of populations, and the functioning and resilience of entire ecosystems (Paine 1966; Polis and Strong 1996; Tinker et al. 2008). There is, for instance, both theoretical and empirical support for the roles of food chain length and primary producer composition in structuring food web architecture, mediating the relationship between species diversity and ecosystem function, and regulating fisheries productivity and biogeochemical fluxes (Post 2002a; Vander Zanden and Fetzer 2007; Young et al. 2013). The ability to quantify food web architecture is critical to understanding ecosystem structure and function, particularly in light of past and future changes in climate and anthropogenic disturbance.

Stable isotope analysis (SIA) has become a widely used tool for examining food web architecture across diverse ecosystems (Layman et al. 2012). This approach is based on the principle that a consumer's bulk carbon (*δ*^13^C) and nitrogen (*δ*^15^N) isotope composition reflects that of its diet coupled with some degree of trophic fractionation (Δ^13^C_C-D_ and Δ^15^N_C-D_) between diet and consumer (Boecklen et al. 2011; McMahon et al. 2013a). While the bulk tissue SIA approach has provided many advances in the fields of trophic and movement ecology (Boecklen et al. 2011; McMahon et al. 2013b), one of the biggest challenges to interpreting bulk stable isotope data is determining whether changes in a consumer's stable isotope values are due to changes in diet/trophic position, variability in trophic fractionation factors, shifts in isotope values at the base of the food web (*δ*^13^C_baseline_, *δ*^15^N_baseline_), or some combination of these factors (Post 2002b; McMahon et al. 2013a,b). Teasing apart the relative influences of trophic and baseline variability on stable isotope values is particularly challenging in complex ecosystems with dynamic baselines and when studying highly mobile predators that feed within multiple isotopically distinct food webs (McMahon et al. 2013b).

Recent advances in the compound-specific stable isotope analysis (CSIA) of individual amino acids (AAs) have allowed for more detailed studies of diet and trophic dynamics that avoid many of the challenges of bulk SIA outlined above (McMahon et al. 2013a; Chikaraishi et al. 2014). For example, the *δ*^13^C values of essential AAs, which animals must acquire directly from their diet (Reeds 2000), show little to no trophic fractionation between diet and consumer (Hare et al. 1991; Howland et al. 2003; McMahon et al. 2010). Further, the high degree of metabolic diversity in essential AA synthesis pathways among primary producers (Hayes 2001; Scott et al. 2006) leads to unique AA *δ*^13^C signatures that get recorded in upper trophic level consumers, virtually unmodified. As a result, essential AA isotope fingerprinting can be used to quantify carbon flow through food webs (Larsen et al. 2009, 2013). In contrast, nonessential AAs can either be *de novo* biosynthesized from a bulk carbon pool or directly routed from dietary protein, with typically highly variable Δ^13^C_C-D_ values across taxa and diet types (Hare et al. 1991; Howland et al. 2003; McMahon et al. 2010). Previous studies suggest that nonessential AA Δ^13^C_C-D_ values are likely related to diet composition and quality, although additional work is needed to understand the underlying mechanisms (McMahon et al. 2010; Newsome et al. 2011).

With respect to *δ*^15^N, individual AAs are commonly divided into trophic and source AAs (after Popp et al. 2007), based on their relative fractionation with trophic transfer (Δ^15^N_C-D_). Trophic AAs, most commonly represented by glutamic acid (Glu), undergo significant isotopic fractionation during transamination/deamination, which often provide greater sensitivity for defining trophic position than bulk SIA (McClelland and Montoya 2002; Chikaraishi et al. 2007, 2009). Conversely, the canonical source AA, phenylalanine (Phe), shows no trophic fractionation between diet and consumer because its metabolic processing does not form or break C-N bonds (McClelland and Montoya 2002; Chikaraishi et al. 2009). Thus, Phe *δ*^15^N provides a proxy for the sources and cycling on nitrogen at the base of food webs (*δ*^15^N_baseline_) (Sherwood et al. 2014; Vokhshoori and McCarthy 2014). Together, the *δ*^15^N value of Glu and Phe can be used to estimate consumer trophic position while accounting for differences in *δ*^15^N_baseline_ without needing to independently characterize and analyze the baseline structure of a food web (McClelland and Montoya 2002; Popp et al. 2007; Chikaraishi et al. 2009). The accuracy of this approach fundamentally depends on the accuracy of the trophic discrimination factor (TDF_Glu-Phe_ = Δ^15^N_Glu_ − Δ^15^N_Phe_) used to calculate trophic position. Accumulating evidence, however, suggests that TDF_Glu-Phe_ values may in fact vary widely across taxa (Germain et al. 2013; Bradley et al. 2014; Hoen et al. 2014; McMahon et al. in press). For instance, Lorrain et al. (2009) hypothesized that AA fractionation may be different in avian consumers because they found that the literature TDF_Glu-Phe_ value of 7.6‰ greatly underestimated the CSIA-based trophic position of wild penguins in their study. Yet, no controlled feeding experiments have examined patterns of AA fractionation in birds.

Our main objective in this study was to examine individual AA *δ*^13^C and *δ*^15^N fractionation patterns for an avian species. We conducted a controlled feeding experiment on captive-reared gentoo penguins (*Pygoscelis papua* Forster 1781) fed a high protein Atlantic herring (*Clupea harengus* Linnaeus 1758) diet. We analyzed feathers because they can be sampled nonlethally, are metabolically inert after synthesis (e.g., Mizutani et al. 1990), and are the most common tissue used in avian SIA studies (Boecklen et al. 2011). Using the newly derived avian-specific AA fractionation factors, we then examined wild penguin foraging ecology and trophic dynamics in the Southern Ocean, including identifying the sources of primary production supporting wild penguins using essential AA *δ*^13^C values and examining how our newly derived avian-specific TDF_Glu-Phe_ values affected estimates of wild penguin trophic position. The results of our study provide new empirical support for the use of CSIA to examine nutritional and foraging ecology, trophic dynamics, and movement ecology of avian consumers.

## Materials and Methods

### Feeding experiment and field collections

A controlled feeding experiment was conducted on 10 captive adult gentoo penguins (five male and five female) at Omaha's Henry Doorly Zoo and Aquarium, Omaha, Nebraska. Penguins were reared on a consistent, high-protein diet of wild-caught Atlantic Herring for 10 months prior to the start of molt. During the study, we hand-fed penguins ad libitum allowing us to record the mass of herring each individual consumed per day during the 30 days prior to molt and during the molt period. Prior to the start of molt, we measured the mass of penguins to the nearest 10 g in order to calculate dietary intake relative to body mass. We calculated the length of the molt period as the number of days between when flippers swell in size and old feathers began to lift and stand out from the body to the end of molt when new body feathers were fully grown. We measured the weight (g) and standard length (mm) of five randomly sampled individual herring per month over the 3 months leading up to and during the molt period (January to March 2008). We collected three newly grown breast feathers from each adult gentoo penguin following their annual molt in late March 2008. During the austral summer of 2010**/**11, we collected breast feathers of five wild adult gentoo penguins (three males, two females) from a colony on King George Island, Antarctica (62°09′S, 58°24′W).

### Sample preparation and analysis

Proximate analysis of crude protein, fat, and carbohydrate content were conducted on herring muscle (*n *=* *3 replicates) at the New Jersey Feed Laboratory, Trenton, New Jersey (AOAC 2005). Amino acid composition (AOAC 2005) of herring and captive penguin feathers was also determined at the New Jersey Feed Laboratory (*n *=* *3 replicates). All feathers were cleaned of surface contaminants using a 2:1 chloroform:methanol rinse. Whole herring samples were homogenized and dried for 48 h in an oven at 60°C. Lipids were extracted from dried herring using a Soxhlet apparatus with a 1:1 petroleum ether/ethyl ether solvent mixture for 8 h (Seminoff et al. 2007). Bulk stable isotope results reported here are from a subset of the samples reported in Polito et al. (2011a).

For CSIA, approximately 3 mg of fish and feather tissue was acid-hydrolyzed in 1 mL of 6 N HCl at 110°C for 20 h to isolate the total free AAs. Samples were evaporated to dryness under a gentle stream of N_2_. The total free AAs were derivatized by esterification with acidified iso-propanol followed by acylation with trifluoroacetic anhydride (Silfer et al. 1991) and brought up in dichloromethane (DCM) for stable isotope analysis. For AA *δ*^13^C analyses, the derivatized AAs were injected on column in split mode at 250°C and separated on a DB-5 column (50 m × 0.5 mm inner diameter; 0.25 *μ*m film thickness; Agilent Technologies, Santa Clara, CA) in a Thermo Trace Ultra gas chromatograph (GC) at the University of California, Santa Cruz, CA. The separated AA peaks were analyzed on a Finnegan MAT Delta^Plus^ XL isotope ratio monitoring mass spectrometer (irm-MS) interfaced to the GC through a GC-C III combustion furnace (960°C) and reduction furnace (630°C). For AA *δ*^15^N analyses, the derivatized AAs were injected on column in splitless mode at 250°C and separated on a BPX5 column (60 m × 0.32 mm inner diameter, 1.0 *μ*m film thickness; SGE Analytical Science, Austin, Texas, USA) in the same CG-C-irm-MS interfaced through a combustion furnace (980°C), reduction furnace (650°C), and a liquid nitrogen trap.

We analyzed the *δ*^13^C and *δ*^15^N values of eleven individual AAs, accounting for approximately 76% and 65% of the total hydrolysable AAs in feathers and herring muscle, respectively. For carbon, we assigned glutamic acid (Glu), aspartic acid (Asp), alanine (Ala), proline (Pro), glycine (Gly), and serine (Ser) as nonessential AAs, and threonine (Thr), leucine (Leu), isoleucine (Ile), valine (Val), and phenylalanine (Phe) as essential AAs. For nitrogen, we assigned Glu, Asp, Ala, Leu, Ile, Pro, Val, Gly, and Ser as trophic AAs, and Phe as the only source AA. Note that threonine (Thr) *δ*^15^N values do no behave similarly to either of these main groups (Hare et al. 1991; McClelland and Montoya 2002; Styring et al. 2010; Germain et al. 2013). Therefore, we have listed Thr as a metabolic AA (according to Germain et al. 2013) and do not discuss Thr *δ*^15^N extensively in this paper. Acid hydrolysis converts glutamine (Gln) and aspartamine (Asn) into Glu and Asp, respectively, due to cleavage of the terminal amine group, resulting in the measurement of combined Gln + Glu (referred to hereby as Glu), and Asn +Asp (referred to hereby as Asp). While some researchers refer to these groupings as Glx and Asx, we chose our terminology here to be consistent with other CSIA studies.

We analyzed 10 individual penguins and three replicate herring samples (one composite sample of five individuals at the beginning, middle, and end of the experiment) from the controlled feeding study, and five individual wild penguins from King George Island, Antarctica. All CSIA samples were analyzed in triplicate along with AA standards of known isotopic composition (Sigma-Aldrich Co., St. Louis, MO, USA). Standardization of runs was achieved using intermittent pulses of a CO_2_ or N_2_ reference gas of known isotopic value. Mean reproducibility of a laboratory algal standard across all individual AAs was ± 0.66‰ for *δ*^13^C and ± 0.34‰ for *δ*^15^N.

### Data analysis

Stable isotope values are expressed in standard delta (*δ*) notation with respect to Vienna Pee Dee Belemnite and air references for *δ*^13^C and *δ*^15^N, respectively. We looked for differences in herring length, weight, and bulk tissue *δ*^13^C and *δ*^15^N values over the three months leading up to and during the molt period with separate one-way analysis of variance (ANOVA) and Tukey's honestly significant difference (HSD) post hoc tests (*α *= 0.05). We compared mean molt periods and consumption of diet as a percent of body mass before and during molt between female and male penguins using separate unpaired two sample t-tests (*n *=* *5 individuals per sex). Trophic fractionation factors (Δ^15^N_C-D_ and Δ^13^C_C-D_) were calculated for feathers as Δ^H^*X*_C-D_ = *δ*^H^X_C_ – *δ*^H^X_D_, where *δ*^H^X_C_ and *δ*^1H^X_D_ represent the *δ*^15^N or *δ*^13^C values of the consumer and diet, respectively. We used separate one-sample t-tests to determine whether bulk tissue and individual AA Δ^13^C_C-D_ and Δ^15^N_C-D_ values were significantly different from zero (*α *= 0.05) (*n *=* *10 individuals). We then compared Δ^13^C_C-D_ and Δ^15^N_C-D_ values among individual AAs with separate one-way ANOVAs and Tukey's HSD post hoc tests (*α *= 0.05) (*n *=* *10 individuals). We tested the hypothesis that diet quality (i.e., AA composition) affected trophic fractionation using linear regressions between AA imbalance (difference in nonessential or trophic AA mol % in diet versus consumer) and trophic fractionation (Δ^13^C_C-D_ or Δ^15^N_C-D_).

We used an isotopic fingerprinting approach (Larsen et al. 2013) to identify the primary producers at the base of the food chain supporting wild gentoo penguins. Briefly, we used published essential AA *δ*^13^C data from eukaryotic microalgae, cyanobacteria, and marine macroalgae (Larsen et al. 2013) as the most likely primary producers supporting penguin food chains in our study. For both the primary producer end members and the wild penguins, individual essential AA *δ*^13^C values were normalized to their means to allow for comparisons of essential AA *δ*^13^C patterns across groups. We identified the most likely primary producer carbon sources contributing to wild penguin essential AA *δ*^13^C patterns using the prediction function of a linear discriminant function analysis (LDA) of normalized essential AA *δ*^13^C values (Thr, Ile, Val, Phe, Leu). For calculating the probability of group membership of the classifier samples, we used a leave-one-out cross-validation approach (Larsen et al. 2009).

We examined how calculation method and TDF_Glu-Phe_ value affected estimated TP_CSIA_ for five species of wild penguins from the Southern Ocean: gentoo penguins (*P. papua*) from this study and northern rockhopper (*Eudyptes chrysocome moseleyi* Mathews & Iredale 1921), southern rockhopper (*E. c. chrysocome* Forster 1781), king (*Aptenodytes patagonicus* Miller 1778), and Adélie (*P. adeliae* Hombron & Jacquinot 1841) penguins from Lorrain et al. (2009). We calculated TP_CSIA_ using the single TDF_Glu-Phe_ approach of Chikaraishi et al. (2009): 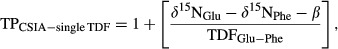
1where *δ*^15^N_Glu_ and *δ*^15^N_Phe_ represent the stable nitrogen isotope values of penguin Glu and Phe, respectively, *β* represents the difference in *δ*^15^N between Glu and Phe of primary producers (3.4‰ for aquatic cyanobacteria and algae [McClelland and Montoya 2002; Chikaraishi et al. 2010]), and TDF_Glu-Phe_ is the literature value of 7.6‰. We also calculated TP_CSIA_ using a multi-TDF_Glu-Phe_ approach that included our newly derived avian-specific TDF_Glu-Phe_ value: 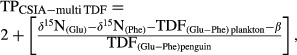
2where TDF_(Glu-Phe)plankton_ = 7.6‰ typical of plankton and other lower trophic level marine organisms (e.g., Chikaraishi et al. 2007), and TDF_(Glu-Phe)penguin_ represents the avian-specific TDF_Glu-Phe_ value derived from this study. We used a two-way ANOVA (species and calculation method as the independent variables and trophic position as the dependent variable) with a Tukey's HSD post hoc test (*α *= 0.05) to compare TP_CSIA_ values of the five species of wild penguins calculated using the single and multi-TDF_Glu-Phe_ equations. All statistics were performed in R version 3.0.2 using RStudio interface version 0.98.501 (R Core Team 2013).

## Results

### Feeding experiment characterization

Penguins in our controlled feeding experiment were fed a herring diet high in protein (72%) and fat (18%) and low in carbohydrates (0.2%) (Table1). Herring length (187.4 ± 4.3 mm; one-way ANOVA, *F*_2,12_ = 0.9, *P *=* *0.43), weight (77.9 ± 6.7 g; one-way ANOVA, *F*_2,12_ = 1.3, *P *=* *0.30), bulk tissue carbon isotope values (−16.9 ± 0.1‰; one-way ANOVA, *F*_2,12_ = 1.1, *P *=* *0.35), and bulk tissue nitrogen isotope values (13.7 ± 0.4‰; one-way ANOVA, *F*_2,12_ = 1.7, *P *=* *0.23) did not change significantly during the course of the feeding study (Table S1). We found notable differences in AA composition between herring muscle (diet) and penguin feather (consumer), particularly for the nonessential AAs proline, glycine, and serine (Table1). Most essential AAs, with the exception of valine (>5% imbalance), had relatively similar molar abundances in the diet and consumer tissues (<2% imbalance) (Table1).

**Table 1:** Mean amino acid molar composition (mol % ± SD) for herring muscle and penguin feather, as well as mean proximate analysis of crude protein, fat, and carbohydrate content (% by weight ± SD) of herring muscle (*n *=* *3). Essential amino acids (carbon) designated with ^E^ and source amino acids (nitrogen) designated with ^S^

	Herring muscle	Penguin feather
Alanine	4.2 ± 0.3	5.2 ± 0.5
Aspartic acid	7.5 ± 0.6	6.4 ± 0.7
Glutamic acid	10.9 ± 0.8	7.1 ± 0.9
Glycine^S^	3.4 ± 0.1	11.5 ± 0.6
Isoleucine^E^	3.1 ± 0.3	4.5 ± 0.4
Leucine^E^	5.9 ± 0.4	7.7 ± 0.6
Phenylalanine^ES^	2.8 ± 0.2	2.1 ± 0.5
Proline	2.7 ± 0.1	10.8 ± 0.4
Serine^S^	2.6 ± 0.2	7.5 ± 0.3
Threonine^E^	3.0 ± 0.2	4.7 ± 0.4
Valine^E^	3.2 ± 0.3	8.7 ± 0.5
Protein	72.4 ± 0.4	–
Fat	18.1 ± 2.1	–
Carbohydrate	0.2 ± 0.1	–

The molt period for penguins in our study ranged from 12 to 18 days (mean 15.0 ± 1.8 days, Table S4) but did not differ between sexes (unpaired two sample *t*-test, *t*_8_ = 0.70, *P *=* *0.51). Penguins consumed 5.2–8.8% (mean 6.9 ± 1.4%) of their body mass in herring per day during the 30 days prior to molt and 0.5–4.0% (mean 1.5 ± 1.0%) of their body mass in herring per day during molt (Table S4). Again, there were no differences between sexes in dietary intake prior to (unpaired two sample *t*-test, *t*_8_ = 0.48, *P* = 0.64) or during molt (unpaired two sample *t*-test, *t*_8_ = 1.13, *P *=* *0.29).

### Carbon isotopes in captive penguins and diet

**Figure 1:**
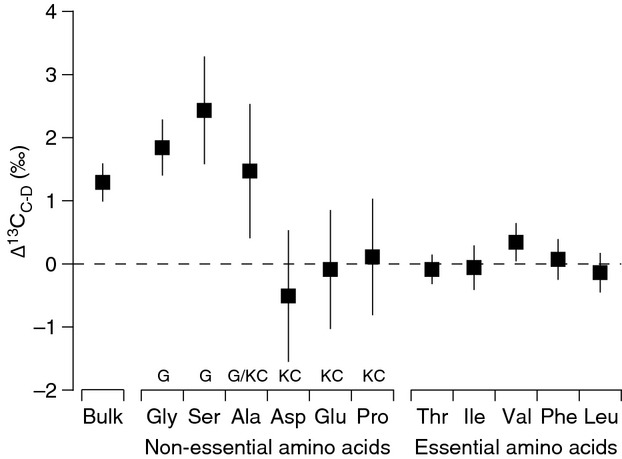
Bulk tissue and individual amino acid stable carbon isotope trophic fractionation (Δ^13^C_C-D_ ± SD, squares) between diet (muscle) and consumer (feather) for gentoo penguins (*Pygoscelis papua*) fed Atlantic herring (*Clupea harengus*). For nonessential amino acids, G = glycolytic pathway synthesis and KC = Kreb cycle synthesis. See methods for amino acid abbreviations (*n *=* *10 individuals).

**Figure 2:**
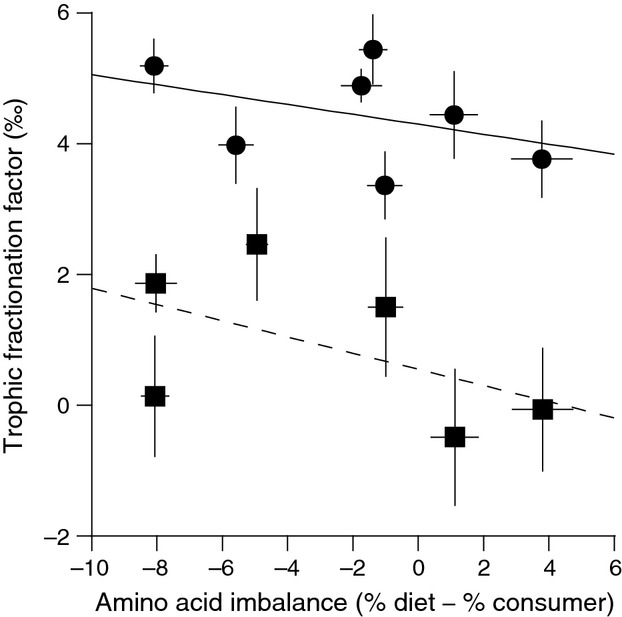
The linear relationship between amino acid imbalance (individual amino acid mol percent abundance in diet minus consumer) and stable carbon isotope trophic fractionation (mean Δ^13^C_C-D_ ± SD; square symbols, solid line) of nonessential amino acids (glutamic acid, aspartic acid, alanine, proline, glycine, and serine) and stable nitrogen isotope trophic fractionation (mean Δ^15^N_C__-D_ ± SD; circle symbols, dashed line) of trophic amino acids (glutamic acid, aspartic acid, alanine, isoleucine, leucine, proline, and valine). Negative values for amino acid imbalance signify a lower mol percent abundance in the diet relative to penguin feather.

Nearly all essential AAs had trophic fractionation values that were not significantly different from 0‰ and were lower than bulk tissue carbon trophic fractionation (Table2, Fig.1). Valine was the only essential AA with a Δ^13^C_C-D_ value significantly greater than 0 (0.3‰, 95% CI = 0.13–0.55‰), although this value is probably not ecologically significant given analytical uncertainly. All nonessential AAs, on the other hand, had highly variable trophic fractionation values (Table2, Fig.1) with significant differences among individual nonessential AAs (one-way ANOVA, *F*_5,54_ = 18.1, *P *=* *1.7e^−10^). The nonessential AAs Gly and Ser, which can be synthesized through glycolytic pathways, and Ala, which can be synthesized via glycolytic and Kreb cycle pathways, had Δ^13^C_C-D_ values that were significantly higher than 0‰ for all individuals (Table2). Conversely, there was far more individual variability in trophic fractionation for the nonessential AAs Glu, Asp, and Pro synthesized via Kreb cycle intermediates (s.d. ∽1.0‰, Table S5), with some individuals having Δ^13^C_C-D_ values greater than 0‰ and others having Δ^13^C_C-D_ values less than 0‰. Despite notable differences in AA abundance between herring muscle and penguin feather (Table1), the slope of the relationship between AA imbalance and nonessential AA Δ^13^C_C-D_ (*f*(*x*) = −0.1*x* + 0.5, *r*^2^* *=* *0.24, *t*_8_ = 0.98, *P *=* *0.35) was not significantly different from 0‰ (Fig.2).

**Table 2:** Mean *δ*^13^C values and *δ*^15^N values (‰ ± SD) in bulk tissue and individual amino acids of herring muscle and penguin feather (*n *=* *3 composite samples for herring and *n *=* *10 individuals for penguins) along with mean Δ^13^C and Δ^15^N values (‰ ± SD) between herring (diet) and penguin (consumer) in the controlled feeding experiment. The results of one sample *t*-tests (*α *= 0.05) to determine whether individual amino acid Δ^13^C_C-D_ and Δ^15^N_C-D_ values were significantly different from 0‰ (*t* statistic and significance [^ns^*P* > 0.05, ^*^P <* *0.05, ^*^^*^P <* *0.01, ^*^^*^^*^P <* *0.001] are in parentheses after the trophic fractionation factors (*n *=* *10 individuals). Essential amino acids (carbon) designated with ^E^ and source amino acids (nitrogen) designated with ^S^

	Herring *δ*^13^C	Penguin *δ*^13^C	Δ^13^C_C-D_ (t statistic)	Herring *δ*^15^N	Penguin *δ*^15^N	Δ^15^N_C-D_ (*t* statistic)
Bulk	−16.9 ± 0.1	−15.9 ± 0.3	1.0 ± 0.3 (10.6^*^^*^^*^)	13.7 ± 0.2	17.2 ± 0.4	3.5 ± 0.4 (27.2^*^^*^^*^)
Alanine	−11.8 ± 0.6	−10.4 ± 1.1	1.5 ± 1.1 (4.4^*^^*^)	21.5 ± 0.5	24.8 ± 0.5	3.4 ± 0.5 (21.3^*^^*^^*^)
Aspartic acid	−12.4 ± 0.8	−12.9 ± 1.0	−0.5 ± 1.0 (−1.6^ns^)	17.5 ± 0.2	21.9 ± 0.6	4.4 ± 0.6 (21.7^*^^*^^*^)
Glutamic acid	−11.9 ± 0.7	−12.0 ± 0.9	−0.1 ± 0.9 (−0.3^ns^)	20.6 ± 0.5	24.4 ± 0.6	3.8 ± 0.6 (20.9^*^^*^^*^)
Glycine	−1.4 ± 0.4	0.4 ± 0.4	1.8 ± 0.4 (13.7^*^^*^^*^)	3.4 ± 0.5	5.3 ± 0.9	1.9 ± 0.9 (6.6^*^^*^^*^)
Isoleucine^E^	−11.6 ± 0.6	−11.7 ± 0.3	−0.1 ± 0.3 (−0.5^ns^)	20.3 ± 0.7	25.7 ± 0.5	5.4 ± 0.5 (33.0^*^^*^^*^)
Leucine^E^	−27.0 ± 0.3	−27.2 ± 0.3	0.2 ± 0.3 (−1.4^ns^)	20.7 ± 0.4	25.6 ± 0.2	4.9 ± 0.2 (64.6^*^^*^^*^)
Phenylalanine^ES^	−24.4 ± 0.4	−24.3 ± 0.3	0.1 ± 0.3 (0.6^ns^)	1.7 ± 0.4	2.0 ± 0.5	0.3 ± 0.5 (1.9^ns^)
Proline	−13.8 ± 0.6	−13.0 ± 0.9	0.1 ± 0.9 (0.4^ns^)	20.7 ± 0.7	25.9 ± 0.4	5.2 ± 0.4 (41.2^*^^*^^*^)
Serine	3.4 ± 0.4	5.9 ± 0.8	2.4 ± 0.8 (9.2^*^^*^^*^)	3.7 ± 0.2	5.6 ± 0.4	1.9 ± 0.4 (13.7^*^^*^^*^)
Threonine^E^	−7.2 ± 0.7	−7.3 ± 0.2	0.1 ± 0.2 (−1.4^ns^)	−10.3 ± 1.4	−21.7 ± 1.1	−11.4 ± 1.1 (−31.7^*^^*^^*^)
Valine^E^	−20.9 ± 0.3	−20.6 ± 0.3	0.3 ± 0.3 (3.6^*^^*^)	22.5 ± 0.3	26.5 ± 0.6	4.0 ± 0.6 (22.1^*^^*^^*^)

### Carbon isotope signatures of wild penguins

**Figure 3:**
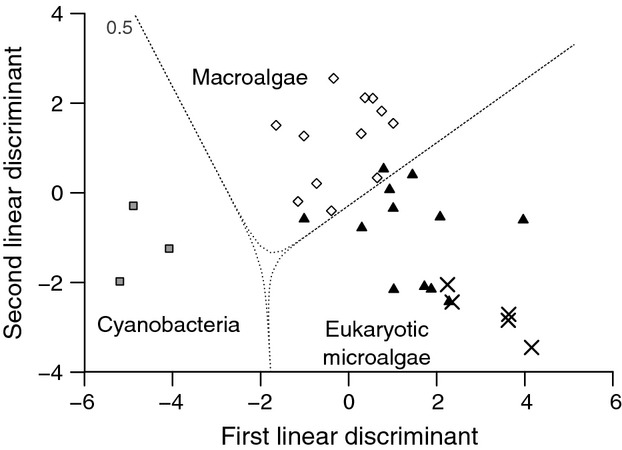
Linear discriminant phylogenetic source analysis based on essential amino acid *δ*^13^C values in wild gentoo penguin feathers from King George Island, Antarctica (solid X, *n *=* *5 individuals). First two linear discriminants are plotted, based on *δ*^13^C values of five essential amino acids (threonine, isoleucine, valine, phenylalanine, and leucine). Normalized data for three primary producer carbon sources, eukaryotic microalgae (filled triangles, *n *=* *12), prokaryotic cyanobacteria (gray squares, *n *=* *3), and marine macroalgae (open diamonds, *n *=* *12) are from Larsen et al. (2013).

Wild-caught penguins around King George Island, Antarctica had essential AA *δ*^13^C patterns (Table3) suggesting that eukaryotic microalgae were the carbon source at the base of the food web supporting penguin production (probability = 99 ± 1%) according to our isotopic fingerprinting LDA model (Fig.3). The model predicted little to no protein contribution from prokaryotic cyanobacteria or benthic macroalgae.

**Table 3:** Mean *δ*^13^C values and *δ*^15^N values (‰ ± SD) in bulk tissue and individual amino acids of wild gentoo penguin feathers (*n* = 5 individuals) collected from King George Island, Antarctica. Essential amino acids (carbon) designated with^E^ and source amino acids (nitrogen) designated with ^S^

	Gentoo *δ*^13^C	Gentoo *δ*^15^N
Bulk	−23.6 ± 0.2	9.8 ± 0.2
Alanine	−20.1 ± 0.7	18.3 ± 0.6
Aspartic acid	−16.2 ± 0.6	14.0 ± 0.7
Glutamic acid	−14.9 ± 1.4	18.9 ± 0.5
Glycine^S^	−10.6 ± 0.5	9.5 ± 0.8
Isoleucine^E^	−14.9 ± 0.9	20.0 ± 0.5
Leucine^E^	−26.3 ± 0.7	19.3 ± 0.6
Phenylalanine^ES^	−23.9 ± 0.9	5.4 ± 0.5
Proline	−17.0 ± 1.2	20.5 ± 0.4
Serine^S^	−7.1 ± 1.2	11.1 ± 0.8
Threonine^E^	−11.7 ± 0.7	−15.5 ± 1.2
Valine^E^	−24.1 ± 0.6	21.1 ± 0.6

### Nitrogen isotopes in captive penguins and diet

**Figure 4:**
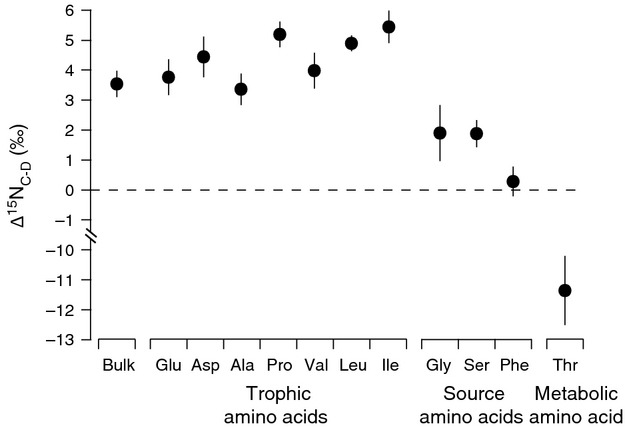
Bulk tissue and individual amino acid stable nitrogen isotope trophic fractionation (Δ^15^N_C__-D_ ± SD, circles) between diet (muscle) and consumer (feather) for gentoo penguins (*Pygoscelis papua*) fed Atlantic herring (*Clupea harengus*). See methods for amino acid abbreviations (*n *=* *10 individuals).

Individual AAs showed a much wider range in AA Δ^15^N_C-D_ values (−11.4‰ to 5.4‰) than was found in bulk tissue Δ^15^N_C-D_ value (Table2; Fig.4). The source AA Phe was the only AA with a Δ^15^N_C-D_ value not significantly different from 0‰ (Table2; Fig.4). All AAs that were a priori identified as trophic AAs as well as two AAs often considered source AAs (Gly, Ser) had Δ^15^N_C-D_ values significantly higher than 0‰ (Table2; Fig.4). There were significant differences in Δ^15^N_C-D_ among individual trophic AAs (one-way ANOVA, *F*_6,63_ = 23.2, *P *=* *2.9e^−14^). However, similar to carbon, the slope of the relationships between AA imbalance and trophic AA Δ^15^N_C-D_ (*f*(*x*) = −0.1*x* + 4.3, *r*^2^ = 0.15, *t*_8_ = 1.1, *P *=* *0.31) was not significantly different from 0‰ despite differences in AA abundance between herring muscle and penguin feather (Fig.2).

### CSIA trophic position calculations of wild penguins

**Figure 5:**
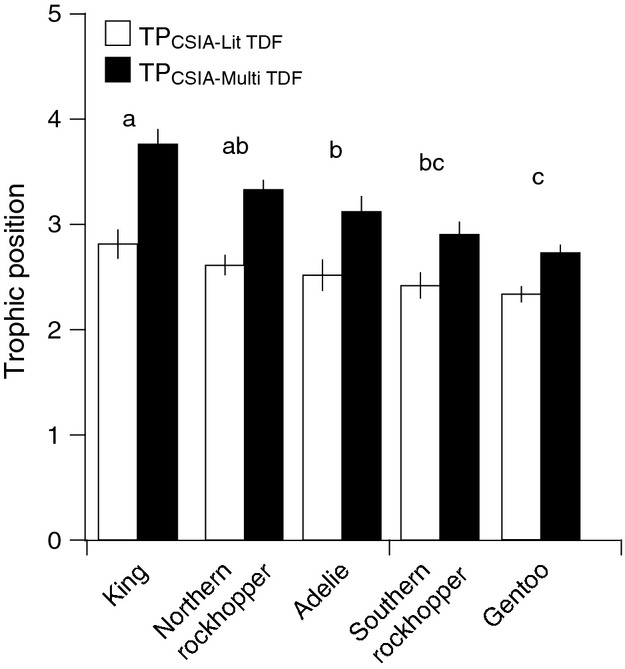
Mean trophic position (± SD) of wild penguins collected from Southern Ocean Islands near Antarctica: king (*n *=* *3), northern rockhopper (*n *=* *2), Adélie (*n *=* *3), southern rockhopper (*n *=* *2), and gentoo (*n *=* *5) penguins. Trophic positions were calculated with a single TDF_G__lu-Phe_ equation using the literature TDF_G__lu-Phe_ of 7.6‰ (Open bars: TP_CSIA__-Lit_ _TDF_) and a multi-TDF_G__lu-Phe_ equation using both the literature and the avian-specific TDF_G__lu-Phe_ values (Black bars: TP_CSIA__-Multi_ _TDF_). Amino acid *δ*^15^N data for king Adélie, and northern and southern rockhopper penguins were from Lorrain et al. (2009). Different letters designated significant differences in trophic position pooled across calculation method according to a two-way ANOVA with Tukey's HSD post hoc test (*P *<* *0.05).

Estimated TP_CSIA_ values of five wild penguin species varied significantly depending on the equation and value of TDF_Glu-Phe_ used (two-way ANOVA, *F*_1,20_ = 86.0, *P *=* *1.1e^−8^) (Fig.5). The TP_CSIA_ values of the five species estimated from the single literature TDF_Glu-Phe_ value of 7.6‰ (eq.1) were significantly lower than estimates from the multi-TDF_Glu-phe_ equation (eq. 2) (Tukey's HSD post hoc test, *P *<* *0.05). There were also significant differen-ces in TP_CSIA_ among the five penguin species (two-way ANOVA, *F*_4,20_ = 18.6, *P *=* *1.6e^−6^) (Fig.5). There was no significant interaction term between species and method of TP_CSIA_ calculation (two-way ANOVA, *F*_4,20_ = 2.6, *P *=* *0.07).

## Discussion

The stable isotope analysis of individual compounds is a powerful and rapidly expanding approach to study food web architecture, including food chain length, resource utilization, and biogeochemical cycling (McMahon et al. 2013a). We found that essential AA *δ*^13^C values and source AA (Phe) *δ*^15^N values in feathers showed little fractionation between diet and consumer, and thus provide excellent proxies of *δ*^13^C_baseline_ and *δ*^15^N_baseline_ in a non lethal, archival tissue that is widely used in avian ecology. Conversely, nonessential AA Δ^13^C_C-D_ values varied significantly according to their biosynthesis pathway and reflected the macromolecule sources being utilized for biosynthesis. When calculating trophic position of wild penguins, we found that using a multi-TDF_Glu-Phe_ equation with our new avian-derived TDF_Glu-Phe_ value (3.5‰) produced more ecologically realistic trophic position estimates than using a single literature value of 7.6‰.

### Carbon trophic fractionation in amino acids

Our results support the general patterns of heterotrophic consumers across a wide range of taxa in marine (McMahon et al. 2010; Newsome et al. 2011) and terrestrial systems (Hare et al. 1991; Howland et al. 2003).We found that modest bulk carbon isotope trophic fractionation was underlain by virtually no trophic fractionation in essential AAs and larger, more variable trophic fractionation in nonessential AAs. The fidelity with which essential AAs reflect dietary sources is one of the major reasons why CSIA is such a powerful tool for dietary reconstruction (McMahon et al. 2013a,b). Here, we demonstrate that the essential AA isotope fingerprinting approach (Larsen et al. 2009, 2013) can be done with nonlethally sampled feathers of wild penguins. Our LDA model predicted that eukaryotic marine microalgae were the dominant primary producers supporting the gentoo penguin food chain around King George Island, Antarctica. This result, while not surprising given that penguins in this region inhabit a diatom and krill-centric pelagic food web (Ducklow et al. 2007), provides a clear demonstration of the potential uses of essential AA *δ*^13^C values in feathers as accurate tracers of baseline primary production sources.

Unlike essential AAs, nonessential AAs showed highly variable trophic fractionation, both among individual nonessential AAs as well as among individual penguins. Nearly all nonessential AAs had nonzero Δ^13^C_C-D_ values when examined at the level of individual penguins, which indicates significant *de novo* biosynthesis of these AAs rather than direct routing from the diet. This was somewhat surprising given that direct isotopic routing of dietary nonessential AAs is energetically favorable when consuming a high-protein diet (Ambrose and Norr 1993; Tieszen and Fagre 1993; Jim et al. 2006). The prevalence of *de novo* biosynthesis may be a function of captive penguins dramatically restricting their food intake during the 2–3 weeks of fasting associated with feather synthesis (Polito et al. 2011a).

During *de novo* biosynthesis, the “scrambled egg” hypothesis assumes that ingested macromolecules (protein, lipids, carbohydrates) are dissembled into a bulk carbon pool and then reassembled into AAs (Martinez del Rio et al. 2009; Newsome et al. 2011). Therefore, we would expect that all individuals feeding on the same diet would have similar Δ^13^C_C-D_ values. However, recent evidence suggests that animals feeding on heterogeneous diets with significant contributions from multiple macromolecules can differentially use the carbon skeletons of these dietary constituents, depending on where in the metabolic process individual AAs are synthesized (O'Brien et al. 2002; McMahon et al. 2010; Newsome et al. 2011). The patterns of nonessential AA trophic fractionation in our study appeared to be related to AA biosynthetic family and the macromolecules used to synthesize them.

The penguins in our study were fed a diet of fish with significant contributions of protein and lipids that differ in their *δ*^13^C values. Lipids are depleted in ^13^C compared to protein, with *δ*^13^C values 4–8‰ lower than whole tissues (DeNiro and Epstein 1977). The glycolytic AAs (Gly, Ser) had consistently positive trophic fractionation values that were significantly greater than the bulk trophic fractionation value. Protein likely provided the ^13^C-enriched source of carbon for the *de novo* biosynthesis of Gly and Ser via 3-phosphoglycerate (Howland et al. 2003; Jim et al. 2006; McMahon et al. 2010). The Kreb cycle AAs (Glu, Asp, Pro), on the other hand, had more variable Δ^13^C_C-D_ values among individual penguins, suggesting greater plasticity in the utilization of different macromolecule carbon sources. For instance, some individual penguins in our study showed positive Kreb cycle AA Δ^13^C_C-D_ values, suggesting *de novo* biosynthesis from a protein carbon source similar to the glycolytic AAs. Conversely, other individuals showed negative Kreb cycle AA Δ^13^C_C-D_ values, suggesting reliance on a different macromolecule pool. The negative Δ^13^C_C-D_ values for Kreb cycle AAs in some individuals suggest that these individuals were using more ^13^C-depleted lipids as the carbon source for biosynthesis. Oxidation of ^13^C-depleted dietary lipids results in the production of ^13^C-depleted acetyl coenzyme A, which is further oxidized in the Kreb cycle to produce ^13^C-depleted keto acids used to synthesize Glu, Asp, and Pro. The enhanced use of lipids in some individuals may indicate nutritional stress during molting. Marine birds store most of their body fuel as fat, primarily triglycerides, which are liberated to free fatty acids to deal with nutritional stress associated with molting and migration (Cherel et al. 1992).

We would expect to see a significant relationship between AA composition and trophic fractionation, where a larger AA imbalance necessitates enhanced biosynthesis and thus larger nonessential AA Δ^13^C_C-D_ values (McMahon et al. 2010). However, utilization of a variety of dietary macromolecules with different *δ*^13^C values provides an explanation for the lack of relationship between AA imbalance and nonessential AA trophic fractionation. Variability in nonessential AA carbon isotope values reflects the complexity in resource utilization among species and individuals, and demonstrates the value of CSIA for examining the underlying drivers of nutritional ecology.

### Nitrogen trophic fractionation in amino acids

A major focus of CSIA in recent years has been the influence of biochemical and physiological processes on consumer stable nitrogen isotope fractionation (McMahon et al. 2013a). Our results generally support the conclusions of previous controlled feeding experiments indicating that bulk trophic fractionation factors (Δ^15^N_C-D_ values = 3.5 ± 0.4‰ in this study) reflect the relatively large fractionation of trophic AAs linked to glutamate metabolism (mean Δ^15^N_C-D_ across all trophic AAs = 4.4 ± 0.8‰ in this study) and no fractionation of the source AA Phe (McClelland and Montoya 2002; Chikaraishi et al. 2009).

The source AA Phe showed minimal fractionation between diet and consumer, preserving a record of *δ*^15^N_baseline_ in feathers. Unlike carbon, where the lack of fractionation of Phe is due to the inability of animals to complete the complex enzymatic pathway necessary to generate the phenol side chain (Gibson and Pittard 1968), minimal nitrogen isotope fractionation is related to C-N bond integrity during the metabolic processing of Phe in animals (e.g., conversion of Phe to tyrosine via phenylalanine 4-monooxidase [Bender 2012; Chikaraishi et al. 2007]). As such, Phe *δ*^15^N values provide a robust tracer of the sources and cycling of N at the base of the food web, independent of the unique synthesis pathways of essential AAs characteristic of distinct primary producer groups (Sherwood et al. 2014; Vokhshoori and McCarthy 2014). Note, Gly and Ser, which have previously been identified as source AAs, exhibited trophic fractionation values significantly larger than 0‰. Our results support the growing body of literature indicating that these AAs should not be considered “source AAs” given their variable Δ^15^N_C-D_ values, from <1‰ to >8‰, across a wide range of species (Chikaraishi et al. 2009; Germain et al. 2013).

While trophic AAs in our study had Δ^15^N_C-D_ values significantly greater than 0‰, as reported in the previous literature (McClelland and Montoya 2002; Chikaraishi et al. 2007, 2009), the magnitude of trophic fractionation for penguins was much lower than reported for many previously reported consumers. Diet quality and consumer's mode of nitrogen excretion have both been hypothesized to affect trophic AA Δ^15^N values (Germain et al. 2013; Bradley et al. 2014; McMahon et al. in press). In a controlled feeding experiment on fishes, McMahon and colleagues (in press) found that Glu Δ^15^N_C-D_ values were significantly related to two important metrics of diet quality, diet protein content and AA imbalance between diet and consumer. Glutamic acid Δ^15^N_C-D_ values were significantly lower for consumers fed higher-quality diets with AA compositions that more closely matched the consumer tissue. In addition, several recent studies have shown low Glu Δ^15^N_C-D_ values for ureotelic/uricotelic/urea-retaining consumers (Germain et al. 2013; Hoen et al. 2014). Excess amino acids are catabolized to ammonia, which is either excreted in the case of most aquatic fishes and invertebrates or converted to urea or uric acid by marine mammals and birds (Balinsky 1972). Glutamate, whose keto acid *α*-ketogluterate is the primary amino acceptor for a number of AAs, plays a lesser role in the excretion of uric acid (Moe 2006). Therefore, the reduction in glutamate transamination among uric acid-producing organisms, such as penguins, may result in a reduction in isotopic fractionation, producing depleted glutamate *δ*^15^N values (Styring et al. 2010). The roles of diet quality and nitrogen excretion mode in determining TDF_Glu-Phe_ values are not mutually exclusive, as both lead to reductions in glutamic acid fractionation during trophic transfers.

### CSIA-based trophic position estimations

The CSIA approach to estimating trophic position has the distinct advantage of providing independent assessment of the number of trophic transfers and *δ*^15^N_baseline_ values for a consumer from a single sample without needing to *a priori* characterize and analyze the baseline of a food web. However, this approach fundamentally relies on accurate TDF_Glu-Phe_ values to produce accurate TP_CSIA_ estimates. Given the minimal fractionation of the source AA Phe coupled with the relatively low trophic fractionation of the trophic AA Glu, we found that the TDF_Glu-Phe_) for penguins (3.5 ± 0.4‰) was significantly lower than the previously reported literature TDF_Glu-Phe_ value of 7.6‰ (Chikaraishi et al. 2007). Our results add to a growing body of literature indicating that TDF_Glu-Phe_ values are not consistently 7.6‰ across all taxa and diet types (Bloomfield et al. 2011; Germain et al. 2013; Bradley et al. 2014; Hoen et al. 2014; McMahon et al. in press). These results likely help explain discrepancies in the previous literature between TP_CSIA_ estimates using a single TDF_Glu-Phe_ value of 7.6‰ and expected trophic positions of upper level marine predators (Lorrain et al. 2009; Dale et al. 2011; Choy et al. 2012).

It is imperative to use appropriate TDF_Glu-Phe_ values to accurately estimate consumer trophic position with CSIA. To illustrate this point, we examined the TP_CSIA_ of five species of wild penguins (king Adélie, and northern and southern rockhopper penguins from Lorrain et al. (2009) and gentoo penguins from this study) that were strictly carnivorous and foraged on both primary and secondary consumers, that is, minimum TP > 3 (Cherel et al. 1993, 2007, 2008; Polito et al. 2011b). TP_CSIA_ estimates of all five species of wild penguins calculated using a single TDF_Glu-Phe_ value of 7.6‰ were less than TP ∽3.0, which was difficult to reconcile with expected trophic positions from bulk SIA and extensive gut content analyses (Cherel et al. 1993, 2007, 2008; Polito et al. 2011b). Furthermore, there were no significant differences in TP_CSIA_ among the five species when calculated with the single TDF_Glu-Phe_ of 7.6‰. Again, this is not ecologically realistic, as king penguins, which typically feed on pelagic fishes (Cherel et al. 1993), should have significantly higher TP_CSIA_ values than Adelie, southern rock hopper, and gentoo penguins, which typically feed on crustaceans (Tremblay and Cherel 2003; Polito et al. 2011b). Conversely, estimates of TP_CSIA_ using a multi-TDF_Glu-Phe_ value that included our avian-specific TDF_Glu-Phe_ provided significantly better agreement with the expected ecological role of penguins in Southern Ocean food webs (Cherel et al. 1993, 2007, 2008; Polito et al. 2011b). However, the multi-TDF_Glu-Phe_ equation still appeared to underestimate penguin trophic positions. This may be because TDF_Glu-Phe_ values are also lower in other trophic levels (e.g., fish) within the penguin food chain (Bradley et al. 2014; Hoen et al. 2014; McMahon et al. in press). Additionally, biases in trophic position estimates from bulk SIA and gut content analyses may account for some of these discrepancies (Layman et al. 2012). None the less, the TDF_Glu-Phe_ values derived from feathers in this study resulted in more realistic TP_CSIA_ estimates for five species wild penguins, four of which were based on previously published AA *δ*^15^N data from blood (Lorrain et al. 2009). This suggests that the avian-specific TDF_Glu-Phe_ values derived from feathers may hold true for other avian tissues as well, although further testing is warranted to confirm such patterns.

In summary, our study provides empirical support for the development and application of CSIA-based studies of avian ecology and illustrates the importance of using appropriate CSIA parameters when characterizing ecosystem properties, such as trophic position and food chain length (DeAngelis et al. 1989; Cabana and Rasmussen 1994; Young et al. 2013).

## Data Accessibility

All data used in this manuscript are present in the manuscript and its supporting material.

## Supporting Information

**Table S1.** Wild-caught Atlantic herring (*Clupea harengus*) fed to gentoo Penguins (*Pygoscelis papua*) prior to and during molt (January to March 2008) at the Omaha's Henry Doorly Zoo and Aquarium, Omaha, Nebraska. **Table S2.** Mean δ^13^C values (‰ ± SD, *n *=* *5 individuals) for bulk tissue and individual amino acids of wild-caught Atlantic herring (*Clupea harengus*) fed to gentoo Penguins (*Pygoscelis papua*) prior to and during molt (January to March 2008) at the Omaha's Henry Doorly Zoo and Aquarium, Omaha, Nebraska. Essential amino acids designated with ^E^. **Table S3.** Mean δ^15^N values (‰ ± SD, *n *=* *5 individuals) for bulk tissue and individual amino acids of wild-caught Atlantic herring (*Clupea harengus*) fed to gentoo Penguins (*Pygoscelis papua*) prior to and during molt (January to March 2008) at the Omaha's Henry Doorly Zoo and Aquarium, Omaha, Nebraska. Source amino acids designated with ^S^. **Table S4.** Individual gentoo penguins (*Pygoscelis papua*) reared at the Omaha's Henry Doorly Zoo and Aquarium, Omaha, Nebraska in 2007/2008. Mass (kg) of penguins was measured just prior to molt. **Table S5.** δ^13^C values (‰) for bulk tissue and individual amino acids from feathers of gentoo penguins fed Atlantic herring at the Omaha's Henry Doorly Zoo and Aquarium, Omaha, Nebraska in 2007/2008. Essential amino acids designated with ^E^. **Table S6.** δ^15^N values (‰) for bulk tissue and individual amino acids from feathers of gentoo penguins fed Atlantic herring at the Omaha's Henry Doorly Zoo and Aquarium, Omaha, Nebraska in 2007/2008. Source amino acids designated with ^S^.
