# Supplementary material for: Carbon and nitrogen isotope fractionation of amino acids in an avian marine predator, the gentoo penguin (Pygoscelis papua)
Source: Ecol Evol. 2015 Feb 25;5(6):1278–90. doi: 10.1002/ece3.1437 (PMC4377271; doi:10.1002/ece3.1437)
Supplement: Supplementary file 1 [file ece30005-1278-sd1.docx]

APPENDIX

Table A1. Wild-caught Atlantic herring (*Clupea harengus*) fed to Gentoo Penguins (*Pygoscelis papua*) prior to and during molt (January to March 2008) at the Omaha’s Henry Doorly Zoo and Aquarium, Omaha, Nebraska.

| ID | Collection  Date | Length  (mm) | Weight  (g) | δ^13^C  (‰) | δ^15^N  (‰) |
| --- | --- | --- | --- | --- | --- |
| ZH30 | 1/2/08 | 190 | 93.0 | -17.1 | 13.7 |
| ZH7 | 1/8/08 | 185 | 59.5 | -16.5 | 14.0 |
| ZH8 | 1/8/08 | 190 | 73.0 | -17.1 | 13.4 |
| ZH5 | 1/14/08 | 174 | 57.6 | -16.8 | 14.1 |
| ZH32 | 1/20/08 | 181 | 73.4 | -16.7 | 14.0 |
| ZH1 | 2/1/08 | 175 | 72.4 | -16.8 | 13.6 |
| ZH2 | 2/1/08 | 195 | 82.5 | -16.9 | 13.9 |
| ZH10 | 2/20/08 | 195 | 88.4 | -16.4 | 13.7 |
| ZH11 | 2/20/08 | 190 | 79.2 | -17.0 | 13.9 |
| ZH17 | 2/25/08 | 175 | 65.7 | -16.6 | 13.8 |
| ZH36 | 3/2/08 | 178 | 71.8 | -16.8 | 13.7 |
| ZH12 | 3/8/08 | 180 | 69.5 | -16.7 | 13.9 |
| ZH13 | 3/8/08 | 200 | 103.5 | -17.7 | 13.8 |
| ZH15 | 3/19/08 | 198 | 97.7 | -18.8 | 12.4 |
| ZH9 | 3/26/08 | 205 | 81.3 | -20.1 | 13.0 |

Table A2. Mean δ^13^C values (‰ ± s.d., *n* = 5 individuals) for bulk tissue and individual amino acids of wild-caught Atlantic herring (*Clupea harengus*) fed to Gentoo Penguins (*Pygoscelis papua*) prior to and during molt (January to March 2008) at the Omaha’s Henry Doorly Zoo and Aquarium, Omaha, Nebraska. Essential amino acids designated with ^E^.

| Herring | January | February | March |
| --- | --- | --- | --- |
| Bulk | -16.9 ± 0.2 | -16.7 ± 0.2 | -17.0 ± 0.4 |
| Glycine | -1.0 ± 0.2 | -1.6 ± 0.1 | -1.6 ± 0.2 |
| Serine | 3.4 ± 0.3 | 3.0 ± 0.4 | 3.8 ± 0.1 |
| Aspartic acid | -11.6 ± 0.2 | -12.4 ± 0.2 | -13.1 ± 0.1 |
| Glutamic acid | -11.8 ± 0.2 | -11.3 ± 0.1 | -12.7 ± 0.1 |
| Proline | -13.3 ± 0.2 | -13.6 ± 0.0 | -14.4 ± 0.3 |
| Alanine | -11.4 ± 0.3 | -11.5 ± 0.2 | -12.6 ± 0.2 |
| Threonine^E^ | -7.4 ± 0.2 | -6.4 ± 0.3 | -7.7 ± 0.2 |
| Isoleucine^E^ | -11.9 ± 0.2 | -11.0 ± 0.4 | -12.0 ± 0.3 |
| Valine^E^ | -20.7 ± 0.1 | -21.2 ± 0.2 | -20.8 ± 0.3 |
| Phenylalanine^E^ | -24.8 ± 0.1 | -24.0 ± 0.2 | -24.4 ± 0.2 |
| Leucine^E^ | -26.7 ± 0.1 | -27.1 ± 0.4 | -27.2 ± 0.2 |

Table A3. Mean δ^15^N values (‰ ± s.d., *n* = 5 individuals) for bulk tissue and individual amino acids of wild-caught Atlantic herring (*Clupea harengus*) fed to Gentoo Penguins (*Pygoscelis papua*) prior to and during molt (January to March 2008) at the Omaha’s Henry Doorly Zoo and Aquarium, Omaha, Nebraska. Source amino acids designated with ^S^.

| Herring | January | February | March |
| --- | --- | --- | --- |
| Bulk | 13.8 ± 0.3 | 13.8 ± 0.1 | 13.4 ± 0.7 |
| Glycine | 3.4 ± 0.7 | 3.9 ± 0.5 | 2.9 ± 0.4 |
| Serine | 3.9 ± 0.2 | 3.5 ± 0.4 | 3.7 ± 0.2 |
| Aspartic acid | 17.5 ± 0.2 | 17.6 ± 0.1 | 17.3 ± 0.1 |
| Glutamic acid | 20.9 ± 0.2 | 20.9 ± 0.2 | 20.0 ± 0.1 |
| Proline | 20.4 ± 0.4 | 21.5 ± 0.6 | 20.2 ± 0.2 |
| Alanine | 22.1 ± 0.4 | 21.3 ± 0.6 | 21.1 ± 0.0 |
| Threonine | -10.2 ± 0.3 | -9.1 ± 0.1 | -11.8 ± 0.1 |
| Isoleucine | 21.1 ± 0.1 | 19.9 ± 0.3 | 19.8 ± 0.7 |
| Valine | 22.8 ± 0.4 | 22.2 ± 0.6 | 22.6 ± 0.6 |
| Phenylalanine^S^ | 1.8 ± 0.2 | 2.0 ± 0.3 | 1.3 ± 0.7 |
| Leucine | 20.8 ± 0.2 | 21.2 ± 0.1 | 20.3 ± 0.1 |

Table A4. Individual Gentoo penguins (*Pygoscelis papua*) reared at the Omaha’s Henry Doorly Zoo and Aquarium, Omaha, Nebraska in 2007/2008. Mass (kg) of penguins was measured just prior to molt. Molt length was calculated as the number of days between when flippers swell in size and old feathers began to lift and stand out from the body to the end of molt when new body feathers were fully-grown. Dietary intake as a percent of penguin body mass (Diet:%BM) was calculated premolt and during molt.

| ISIS# | Sex | Mass  (kg) | Molt Length  (days) | % Diet:BM  Premolt | % Diet:BM  Molt |
| --- | --- | --- | --- | --- | --- |
| 8244 | Female | 4.57 | 15 | 8.6 | 0.5 |
| 8248 | Female | 4.83 | 15 | 5.3 | 2.0 |
| 8249 | Male | 5.38 | 13 | 5.2 | 1.0 |
| 8254 | Female | 4.93 | 17 | 7.5 | 0.6 |
| 9487 | Female | 5.96 | 14 | 6.8 | 1.1 |
| 9490 | Male | 7.48 | 12 | 6.6 | 4.0 |
| 13251 | Male | 6.21 | 15 | 8.8 | 1.1 |
| 14187 | Female | 6.54 | 16 | 5.2 | 1.5 |
| 15108 | Male | 7.01 | 15 | 6.9 | 1.9 |
| 16814 | Male | 7.18 | 18 | 8.1 | 1.1 |

Table A5. δ^13^C values (‰) for bulk tissue and individual amino acids from feathers of Gentoo penguins fed Atlantic herring at the Omaha’s Henry Doorly Zoo and Aquarium, Omaha, Nebraska in 2007/2008. Essential amino acids designated with ^E^.

| ISIS# | Bulk | Gly | Ser | Asp | Glu | Pro | Ala | Thr^E^ | Ile^E^ | Val^E^ | Phe^E^ | Leu^E^ |
| --- | --- | --- | --- | --- | --- | --- | --- | --- | --- | --- | --- | --- |
| 9490 | -16.0 | 0.3 | 5.9 | -14.1 | -12.8 | -14.6 | -11.7 | -7.4 | -11.6 | -20.7 | -24.2 | -27.5 |
| 8244 | -15.6 | 1.0 | 6.9 | -11.9 | -11.3 | -13.1 | -9.3 | -7.1 | -11.4 | -20.4 | -24.0 | -26.9 |
| 8254 | -16.2 | 0.2 | 4.9 | -14.0 | -13.2 | -14.6 | -11.7 | -7.1 | -12.1 | -20.7 | -24.4 | -27.4 |
| 8248 | -15.9 | 0.7 | 6.2 | -13.9 | -12.8 | -14.1 | -10.6 | -7.1 | -11.7 | -20.1 | -24.1 | -27.0 |
| 8249 | -15.8 | 0.8 | 6.0 | -12.0 | -11.1 | -13.3 | -9.9 | -7.3 | -11.5 | -20.5 | -24.1 | -26.5 |
| 9487 | -16.0 | 0.4 | 5.6 | -12.5 | -11.4 | -13.6 | -10.3 | -7.1 | -11.6 | -20.8 | -24.2 | -27.3 |
| 15108 | -15.5 | 0.5 | 6.8 | -12.1 | -11.4 | -12.2 | -9.3 | -7.4 | -11.5 | -20.7 | -25.0 | -27.3 |
| 13251 | -15.6 | 1.0 | 6.7 | -11.3 | -10.7 | -12.3 | -8.7 | -7.0 | -11.1 | -20.1 | -24.3 | -26.9 |
| 16814 | -16.3 | -0.4 | 4.4 | -13.6 | -12.7 | -14.4 | -11.2 | -7.6 | -12.2 | -21.0 | -24.4 | -27.3 |
| 14187 | -16.3 | 0.0 | 5.2 | -13.4 | -13.0 | -14.3 | -11.1 | -7.6 | -12.1 | -20.8 | -24.7 | -27.5 |

Table A6. δ^15^N values (‰) for bulk tissue and individual amino acids from feathers of Gentoo penguins fed Atlantic herring at the Omaha’s Henry Doorly Zoo and Aquarium, Omaha, Nebraska in 2007/2008. Source amino acids designated with ^S^.

| ISIS# | Bulk | Gly | Ser | Asp | Glu | Pro | Ala | Thr | Ile | Val | Phe^S^ | Leu |
| --- | --- | --- | --- | --- | --- | --- | --- | --- | --- | --- | --- | --- |
| 9490 | 18.0 | 5.4 | 5.6 | 21.5 | 24.6 | 25.7 | 24.4 | -22.4 | 25.8 | 27.1 | 1.9 | 25.6 |
| 8244 | 17.5 | 6.2 | 5.5 | 22.8 | 25.2 | 25.9 | 24.4 | -23.7 | 25.8 | 26.2 | 2.6 | 25.8 |
| 8254 | 17.2 | 6.1 | 5.9 | 22.5 | 24.8 | 26.6 | 26.1 | -21.1 | 26.2 | 26.3 | 2.2 | 26.0 |
| 8248 | 16.7 | 5.5 | 5.4 | 22.1 | 24.9 | 25.6 | 24.7 | -22.7 | 25.1 | 26.6 | 1.8 | 25.7 |
| 8249 | 17.4 | 5.6 | 5.8 | 21.8 | 24.3 | 25.5 | 24.8 | -22.5 | 25.8 | 27.4 | 2.3 | 25.8 |
| 9487 | 17.1 | 5.7 | 5.5 | 22.7 | 24.7 | 26.1 | 24.7 | -21.0 | 25.5 | 25.8 | 2.2 | 25.8 |
| 15108 | 17.1 | 5.8 | 5.2 | 21.2 | 24.0 | 25.3 | 24.7 | -20.8 | 25.1 | 25.9 | 2.1 | 25.3 |
| 13251 | 17.7 | 5.3 | 5.2 | 22.2 | 24.0 | 26.1 | 25.1 | -22.1 | 25.3 | 26.0 | 2.3 | 25.4 |
| 16814 | 16.8 | 3.6 | 6.6 | 20.9 | 23.8 | 25.7 | 24.8 | -20.4 | 25.8 | 26.7 | 1.1 | 25.3 |
| 14187 | 16.9 | 3.7 | 5.1 | 21.4 | 23.3 | 26.3 | 24.8 | -20.4 | 26.8 | 27.1 | 1.3 | 25.6 |
